# Intraoperative intraocular pressure changes during robot-assisted radical prostatectomy: associations with perioperative and clinicopathological factors

**DOI:** 10.1186/s12894-020-00595-5

**Published:** 2020-03-12

**Authors:** Yuko Shirono, Itsuhiro Takizawa, Takashi Kasahara, Ryo Maruyama, Kazutoshi Yamana, Toshiki Tanikawa, Noboru Hara, Yuta Sakaue, Tetsuya Togano, Tsutomu Nishiyama, Takeo Fukuchi, Yoshihiko Tomita

**Affiliations:** 1 Division of Urology, Department of Regenerative and Transplant Medicine, Graduate School of Medical and Dental Sciences, Niigata University, Asahimachi 1, Niigata, 951-8510 Japan; 2 Division of ophthalmology and Visual Science, Graduate School of Medical and Dental Sciences, Niigata University, Niigata, Japan

**Keywords:** Intraoperative intraocular pressure, Non-glaucoma, Robot-assisted radical prostatectomy, Steep Trendelenburg position

## Abstract

**Background:**

Steep Trendelenburg position (ST) during robot-assisted radical prostatectomy (RARP) poses a risk of increase in intraocular pressure (IOP) in men receiving robot-assisted radical prostatectomy (RARP). The aim of the study was to identify clinicopathological factors associated with increased IOP during RARP.

**Methods:**

We prospectively studied 59 consecutive prostate cancer patients without glaucoma. IOP was measured at 6 predefined time points before, during and after the operation (T1 to T6).

**Results:**

Compared with T1, IOP decreased after beginning of anesthesia(T2) (by − 6.5 mmHg, *p* < 0.05), and increased 1 h after induction of pneumoperitoneum in the steep Trendelenburg position (ST) (T3) (+ 7.3 mmHg, *p* < 0.05). IOP continued to increase until the end of ST (T4) (+ 10.2 mmHg, p < 0.05), and declined when the patient was returned to supine position under general anesthesia (T5) (T1: 20.0 and T5: 20.1 mmHg, p above 0.05). The console time affected the elevation of IOP in ST; IOP elevation during ST was more prominent in men with a console time of ≥4 h (*n* = 39) than in those with a console time of < 4 h (*n* = 19) (19.8 ± 6.3 and 15.4 ± 5.8 mmHg, respectively, *p* < 0.05). Of the 59 patients, 29 had a high baseline IOP (20.0 mmHg or higher), and their IOP elevated during ST was also reduced at T5 (T1: 22.6 and T5: 21.7 mmHg, p above 0.05). There were no postoperative ocular complications.

**Conclusions:**

Console time of < 4 h is important to prevent extreme elevation of IOP during RARP. Without long console time, RARP may be safely performed in those with relatively high baseline IOP.

## Background

Robot-assisted radical prostatectomy (RARP) is generally contraindicated in patients with glaucoma. In a case that developed vision disorder after RARP, it was considered that this was caused by the increase in intraocular pressure (IOP) during surgery due to the prolonged use of the steep Trendelenburg position (ST). Additionally, perioperative ischemic optic neuropathy is prevalent in older patients with a high preoperative IOP [1].

At present, many men with high baseline IOP cannot benefit from RARP. However, the identification of intraoperative factors associated with the increase of IOP during ST may lead to feasibility of RARP in them without compromising safety [2]. Also, robot-assisted surgery for other disorders such as advanced bladder cancer is expected to require a longer operation time than RARP, and the situation with the expansion of robot-assisted surgery possibly warrants studies on treatment-related adverse events represented by the increase in IOP and the factors thereof. We therefore examined the changes in IOP in men receiving RARP to identify disease-specific, technical, and operator-dependent factors associated with increased IOP during perioperative period.

## Methods

The study was prospectively designed, and this research project was approved by the Ethics Committee of our institution. The primary endpoint was to survey the incidence of the high perioperative IOP in men receiving RARP, and the secondary endpoint was to identify clinical factors associated with perioperative IOP.

### Patients

Fifty-nine consecutive patients with prostate cancer without corneal disease or glaucoma, who underwent RARP at Niigata University Medical and Dental Hospital between March 2014 and February 2016, were enrolled in this study. Written informed consent was obtained from all patients. Surgical procedures were performed by experienced urologists (TK, TN, and YT).

### Surgery and measurement of intraocular pressure

General anesthesia was conducted with intravenous anesthesia (using propofol) in 15 cases, and by inhalation anesthesia with sevoflurane in 4 cases, and with desflurane in 35 cases. During RARP, IOP was measured at 6 predefined time points: T1: prior to the induction of anesthesia; T2: anesthetized and supine; T3: 1 h after induction of pneumoperitoneum in the ST position; T4: while in pneumoperitoneum, at the end of ST; T5: anesthetized supine before awakening; T6: 30 min after recovery from anesthesia, while still supine. Bilateral IOP was measured using a hand-held tonometer (Tono-Pen, Reichert Technologies, Depew, NY, USA) [3]. Mean IOP was calculated for one eye based on those measured 3 times with inter-assay coefficients of variability less than 10%, and the average IOP of the right and left eyes was applied for comparison and analysis.

Patients were also evaluated by experienced ophthalmologists (YS and TT) before surgery, within 2 weeks of RARP, and again at 3 months after surgery. Any visual symptoms were surveyed thereafter (observation period, median 24 months).

### Statistical analysis

For statistical analysis, the chi-square, paired t-, and Mann-Whitney U tests were used, and a *p* value less than 0.05 was considered significant. Statistical analyses were calculated and tested using SPSS software ver. 16.0 (SPSS, Inc., Chicago, IL).

## Results

Postoperative complications included port site hemorrhage in 1 case, compartment syndrome (Grade 2) in 1 case, abdominal incisional hernia in 2 cases, lymphorrhea (Grade 1) in 1 case, anastomotic leak and peritonitis in 1 case, anastomotic leak and pelvic hemorrhage in 1 case, bladder tamponade and urethral stricture in 1 case, and drug-induced liver injury (Grade 2) in 1 case [4]. There was no peri- and post-operative ocular complication.

Patient characteristics and perioperative outcomes are shown in Table 1. Preoperative serum PSA levels ranged between 2.8 and 33.0 (median: 8.0) ng/dl. Biopsy Gleason score was 6 or less, 7, and 8 or higher in 8 (13.6%), 30 (50.8%), and 21 (35.6%) men, respectively. The clinical T stage was T1c, T2, and T3 in 35 (59.3%), 12 (22.0%), and 1 (1.7%), respectively. The operation time ranged between 129 and 487 (median: 265) min, and console time between 88 and 429 (median: 207) min. Unilateral nerve-sparing technique was performed in 11 (18.6%), and bilateral nerve-sparing was conducted in 2 (3.4%) patients.

**Table 1:** Patients’ demographics and perioperative outcomes

Variables	n = 59
Age [y.o.], median (range)	65.0 (51–74)
Body weight [kg], median (range)	65 (50.0–92.2)
Body mass index [kg/m^2^], median (range)	23.0 (18.9–30.9)
Prostate volume [cm^3^], median (range)	24.0 (15.0–57.0)
Serum PSA [ng/dl], median (range)	8.0 (2.8–33.0)
Biopsy Gleason score, n (%)	
6 or less	8 (13.6)
7	30 (50.8)
8 or higher	21 (35.6)
clinical T stage, n (%)	
T1c	35 (59.3)
T2a	8 (13.6)
T2b	3 (5.1)
T2c	2 (3.4)
T3	1 (1.7)
Unknown	10 (16.9)
Operation time [min], median (range)	265 (129–487)
Console time [min], median (range)	207 (88–429)
Nerve-sparing, n (%)	
Unilateral	11 (18.6)
Bilateral	2 (3.4)
None	46 (80.0)
Intraoperative blood loss, median (range)	275 (0–1650)

**Fig. 1:**
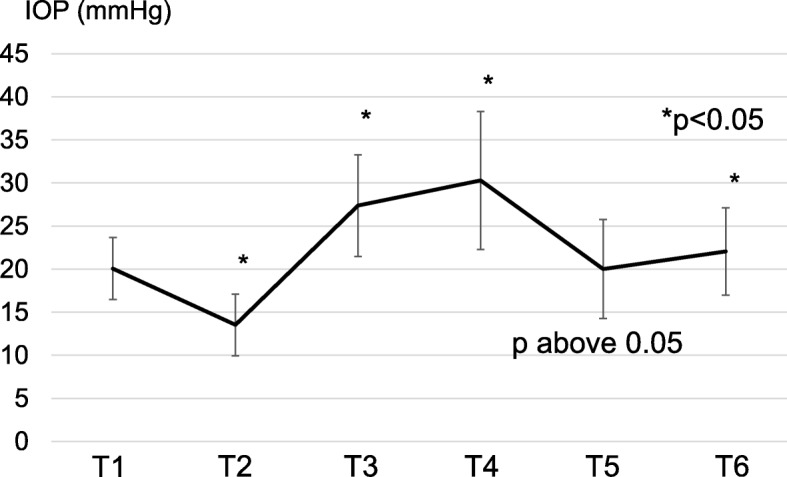
Intraoperative intraocular pressure (IOP) changes in non-glaucoma patients (*n* = 59). IOP was measured at 6 predefined time points: T1: prior to introduction of anesthesia; T2: anesthetized and supine; T3: 1 h after induction of pneumoperitoneum in steep Trendelenburg position (ST); T4: while in pneumoperitoneum, at the end of ST; T5: returned to supine position under anesthesia; T6: 30 min after end of anesthesia, while still supine. IOP at T1 was used as the reference

Intraoperative IOP change was shown in Fig. 1. IOP at T2 to T6 was compared with preoperative baseline IOP (T1). IOP decreased after the induction of anesthesia (T1 to T2: by mean − 6.5 mmHg, *p* < 0.05) and increased 1 h after induction of pneumoperitoneum in ST (T3) (T1 to T3: by mean + 7.3 mmHg, p < 0.05). IOP also increased in a time-dependent manner until the end of ST (T1 to T4: by mean + 10.2 mmHg, p < 0.05), and after console surgery when the patient was returned to supine position under anesthesia (T5). IOP recovered to the baseline level after the operation (T1: 20.0 mmHg and T5: 20.1 mmHg, p above 0.05). At 30 min after the end of anesthesia (T6), IOP slightly increased again compared with that at T1 (T1 to T6: by mean + 2.0 mmHg).

Of the 59 patients, 29 had a high baseline IOP (20.0 mmHg or higher). Their IOP at T1 ranged between 20.0 and 27.3 (mean: 22.6) mmHg; it was elevated during ST(T1 to T4: by mean + 9.9 mmHg, *p* < 0.05), and was also reduced at T5 (range: 14.5 and 37.3, mean 21.7 mmHg, p above 0.05). Their IOP at T6 also increased slightly (mean 23.6 mmHg), but it was not different compared with IOP at T1 (p above 0.05).

**Fig. 2:**
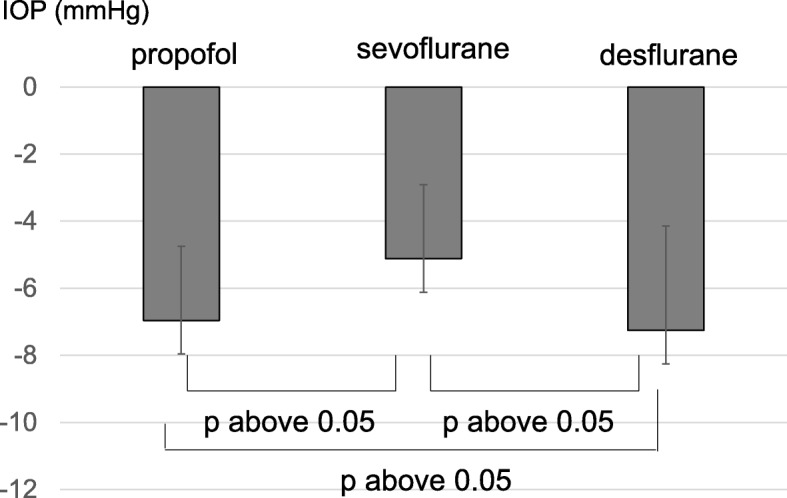
Intraoperative intraocular pressure (IOP) changes according to the type of anesthesia used. The type of anesthesia had no effect on intraoperative intraocular pressure changes

We further explored the factors that affected IOP change. Effect of the type of applied anesthesia was first examined, since IOP was reduced after the introduction of anesthesia (T2) (Fig. 1). There was no significant difference in IOP reduction according to the type of anesthesia used (p above 0.05) (Fig. 2); propofol resulted in − 7.0 ± 2.2 mmHg (mean ± standard deviation), sevoflurane in − 5.1 ± 2.2 mmHg, and desflurane in − 7.3 ± 3.1 mmHg change in IOP.

**Fig. 3:**
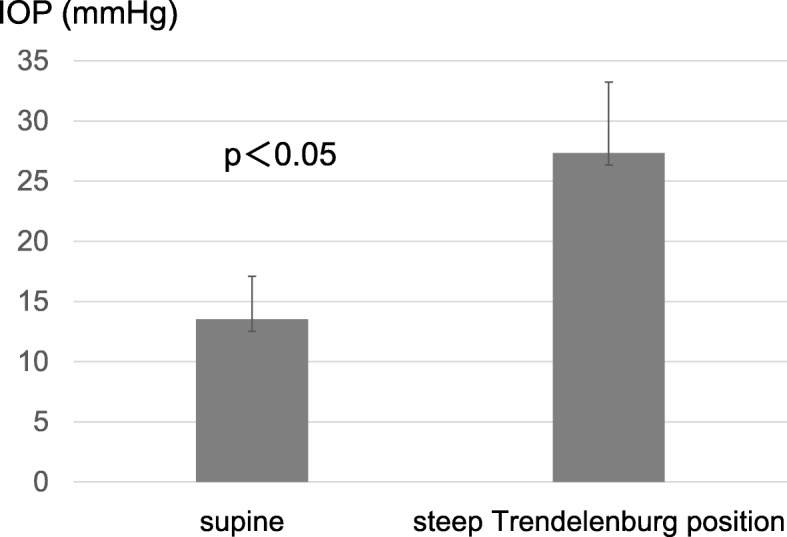
Increased intraocular pressure (IOP) at 1-h console time (T3). Steep Trendelenburg position led to increased intraocular pressure (IOP) compared with that during anesthetized supine position (T2)

IOP was 13.5 ± 3.6 mmHg in the supine position under anesthesia (T2), and was 27.3 ± 5.9 mmHg in the ST at 1 h of console operation (T3) (Fig. 1); thus, ST caused significant increase in IOP (*p* < 0.05) (Fig. 3). Age, body weight, and BMI were not associated with the increase in IOP during ST (p above 0.05 respectively).

**Fig. 4:**
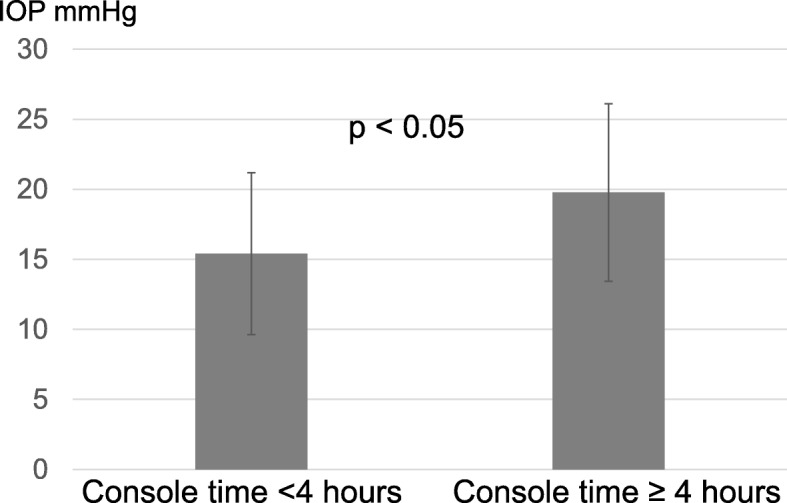
The association of console time with the increase in IOP. The difference of IOP between at T2 and T4 was 15.4 ± 5.8 mmHg in men with a console time of < 4 h (*n* = 19); it was 19.8 ± 6.3 mmHg in those with a console time of ≥4 h (*n* = 39) (*p* < 0.05)

The longer console time was associated with further increase in IOP during RARP; the difference of IOP between T2 and T4 was 15.4 ± 5.8 mmHg in men with a console time of < 4 h (*n* = 19), whereas it was 19.8 ± 6.3 mmHg in those with a console time of ≥4 h (*n* = 39) (*p* < 0.05) (Fig. 4). There was no significant difference between the 2 groups concerning age (p above 0.05), body weight (p above 0.05), BMI (p above 0.05), or prostate volume (p above 0.05).

We additionally analyzed factors associated with a longer operation time. There was a difference between nerve-sparing (median 245, range 186–329 min) and non-nerve-sparing prostatectomies (median 196, 88–429 min) (*p* < 0.05), biopsy Gleason score of 7 or less (median 197, range 92–262 min) and 8 or higher (median 214, range 88–429) (p < 0.05), and low- to intermediate- (median 194, range 113–329 min) and high-risk (median 223, range 88–429) (p < 0.05) prostate cancers according to the D’Amico risk classification [5]. There was no significant difference in terms of body weight, cT stage, prostate volume, intraoperative blood loss, PSA, and a history of prior abdominal surgery.

## Discussion

In the present study, IOP increased when patients were in ST (T3 to T4), and it was thereafter elevated in a time-dependent manner during ST. When ST was changed to supine position (T5), IOP recovered to the level similar to that at baseline (T1), although it was slightly elevated again at T6 most probably due to the withdrawal of anesthesia. In addition, we found that the console time significantly affected the increase in IOP during RARP at a cut-off of 4 h.

There is another report on the effect of ST on IOP during robotic surgery including 43 RALP cases [6]. The authors found that the highest IOP was at the end of ST under pneumoperitoneum which is in perfect agreement with our findings.

Recognized risk factors for glaucoma include high IOP, older age, family history, ethnicity (African descent), hypotension, thin central corneal pressure, myopia, and diabetes [3, 7]. With lower ocular perfusion pressure, degenerative dropout of retinal ganglion cells causes optic nerve damage [7]. High IOP is found in 4–10% of individuals aged more than 40 years [8], and in these patients the onset rate of open angle glaucoma is increased 10–15-fold [9]. Preoperative eye examination is essential for elderly patients, who are affected by glaucoma.

Increased central venous pressure, pneumoperitoneum with carbon dioxide, and increased airway pressure have been proposed [2, 10] as mechanisms affecting IOP elevation in patients in the ST. Increased central venous pressure due to ST raises the upper scleral venous pressure and suppresses the outflow of aqueous humor. With pneumoperitoneum, increased amounts of carbon dioxide dissolve in the blood and decrease the ventilation volume due to compression of the diaphragm, causing hypercapnemia, and the IOP rises because the choroidal vascular volume increases due to vasodilation. Excessive increase in airway pressure due to ST raises intrathoracic pressure and thereby increases central venous pressure [10].

Although a direct causal relationship between elevated IOP and postoperative visual dysfunction has not been clarified to date, it has been reported that transient focal visual field defects occur in 28% of non-glaucoma patients after RARP surgery [2]; permanent ischemic optic neuropathy has occurred in 1 case after RARP, and in 1 case after laparoscopic radical prostatectomy [1]. Therefore, in terms of IOP, it can be inferred that the anesthesia used for laparoscopic surgery (propofol) can reduce the burden on the optic nerve. In addition, there have been several reports that propofol can decrease IOP and that eye perfusion pressure can be maintained higher when using propofol than when using sevoflurane or desflurane (inhalation anesthesia), by decreasing the effect on intraocular muscle tone [11–13] In this study, 15 cases were anesthetized using propofol, 4 cases using sevoflurane, and 35 cases using desflurane; the agent used did not result in statistically significant differences in IOP.

The current study showed that a console time of < 4 h did not lead to a marked increase in IOP during RARP. Although it involved apparently non-glaucomatous subjects, the present patient series included 29 elderly patients, who had high IOP at baseline (20 mmHg or higher) [3, 9]. Their IOP increased during ST, but was reduced to baseline levels at T5 (mean 21.7 mmHg, p above 0.05) and T6 (mean 23.6 mmHg, p above 0.05). Also, no postoperative ocular complications were observed in these patients. To the best of our knowledge, perioperative IOP has barely been studied in men with high baseline IOP receiving RARP. Without a long console time, thus, the use of RARP may be expanded to such men without compromising safety.

## Conclusions

To prevent a marked elevation of IOP in men undergoing RARP, a console time of < 4 h is important. Men with moderately high baseline IOP also received this procedure without ocular complications. Without a long console time, the use of RARP may be expanded to men having a high baseline IOP without compromising safety, and further studies are thus warranted.

## Data Availability

The datasets used and/or analysed during the current study are available from the corresponding author on reasonable request. The relevant information is added to the manuscript.

## Abbreviations

IOP: Intraocular pressure
RARP: Robot-assisted radical prostatectomy
ST: Steep Trendelenburg position
